# Population genomic analysis of clinical ST15 *Klebsiella pneumoniae* strains in China

**DOI:** 10.3389/fmicb.2023.1272173

**Published:** 2023-11-15

**Authors:** Li Feng, Mingcheng Zhang, Zhiyi Fan

**Affiliations:** Jiyang College, Zhejiang A&F University, Zhuji, China

**Keywords:** *Klebsiella pneumoniae*, carbapenemase, molecular epidemiology, genomics, phylogenetics, pan-genome

## Abstract

ST15 *Klebsiella pneumoniae* (Kpn) is a growing public health concern in China and worldwide, yet its genomic and evolutionary dynamics in this region remain poorly understood. This study comprehensively elucidates the population genomics of ST15 Kpn in China by analyzing 287 publicly available genomes. The proportion of the genomes increased sharply from 2012 to 2021, and 92.3% of them were collected from the Yangtze River Delta (YRD) region of eastern China. Carbapenemase genes, including OXA-232, KPC-2, and NDM, were detected in 91.6% of the studied genomes, and 69.2% of which were multidrug resistant (MDR) and hypervirulent (hv). Phylogenetic analysis revealed four clades, C1 (KL112, 59.2%), C2 (mainly KL19, 30.7%), C3 (KL48, 0.7%) and C4 (KL24, 9.4%). C1 appeared in 2007 and was OXA-232-producing and hv; C2 and C4 appeared between 2005 and 2007, and both were KPC-2-producing but with different levels of virulence. Transmission clustering detected 86.1% (n = 247) of the enrolled strains were grouped into 55 clusters (2–159 strains) and C1 was more transmissible than others. Plasmid profiling revealed 88 plasmid clusters (PCs) that were highly heterogeneous both between and within clades. 60.2% (*n* = 53) of the PCs carrying AMR genes and 7 of which also harbored VFs. KPC-2, NDM and OXA-232 were distributed across 14, 4 and 1 PCs, respectively. The MDR-hv strains all carried one of two homologous PCs encoding *iucABCD* and *rmpA2* genes. Pangenome analysis revealed two major coinciding accessory components predominantly located on plasmids. One component, associated with KPC-2, encompassed 15 additional AMR genes, while the other, linked to OXA-232, involved seven more AMR genes. This study provides essential insights into the genomic evolution of the high-risk ST15 CP-Kpn strains in China and warrants rigorous monitoring.

## 1. Introduction

*Klebsiella pneumoniae (Kpn)* causes a range of infections, including pulmonary, urinary tract, bloodstream, and surgical site infections (Paczosa and Mecsas, 2016). Carbapenems are commonly employed for treating severe infections caused by multidrug-resistant (MDR) *Enterobacteriaceae*, including *AmpC β*-lactamases and extended-spectrum *β*-lactamases (ESBLs). Unfortunately, the extensive use of carbapenems in recent years has expedited the emergence of resistant strains (Shrivastava et al., 2018).

As in many other countries, Kpn is a notifiable disease in China (Zhang et al., 2017; Kazmierczak et al., 2021; Lee et al., 2022). According to the China Antimicrobial Surveillance Network (CHINET) results, although the resistance rate of Kpn to carbapenems showed a steady downward trend from 2018 to 2021, the detection rate was still over 23% (Hu et al., 2018). The detection rate reached more than 30% in some areas, and the trend is slowly increasing. Previous epidemiology studies have shown that KPC-2 is the widest disseminated carbapenemase in China, and the dominant ST is ST11 (Zhang et al., 2017). However, ST15 Kpn becomes an emerging high-risk clone with frequent hospital outbreaks (Cienfuegos-Gallet et al., 2022). A multi-center study showed a shift in the dominant sequence type of carbapenemase-producing Kpn (CP-Kpn) bloodstream infections from ST11 to ST15 in northeast China, especially after the COVID-19 pandemic (Chen J. et al., 2021). ST15 Kpn has been reported to contain the *bla*_OXA-232_ gene situated within the ColKP3-type (also known as ColE-type) plasmid in China (Yin et al., 2017; Shu et al., 2019; Chen Y. et al., 2021). Long-term nosocomial surveillance of OXA-48-like carbapenemases report in Zhejiang province, southeast China, from 2018 to 2021 showed that ST15 CP-Kpn isolates are the primary carriers in recent years (Zhang et al., 2023).

In addition to threats from CP-Kpn, infections due to hypervirulent Kpn have steadily increased over the last three decades (Russo and Marr, 2019). The hypervirulent strains are usually isolated from community-acquired infections and may cause a liver abscess, bloodstream infection, or meningitis, among other pathological conditions (Choby et al., 2020). The reported best-characterized virulence factors with experimental support for conveying the hypervirulent phenotype, including *iuc*, *iro*, *rmpA*, and *rmpA2*, are encoded by genes on hypervirulent (hv) plasmids (Zhu J. et al., 2021). Increasing occurrence of multidrug resistance (MDR) and hv Kpn (MDR-hvKpn) convergent clones is being observed(Wyres et al., 2020b). A public health concern is that virulence gene carriage has been reported to be 34.2% for CP-Kpn in China (Zhang et al., 2020).

Recent advancements in the whole-genome sequencing and extended applications of bioinformatic tools facilitate gathering information on thousands of bacterial species on their virulence factors (VF), antimicrobial resistance (AMR), and genetic relationship (Schürch et al., 2018). Comparative genomics of microbial genomes assists in understanding the genomic variations, the basis of diverse phenotypes (Wyres et al., 2020a). Although there has been a recent phylogenomics study on ST15 Kpn strains worldwide, only 9 strains were isolated from China (Rodrigues et al., 2023).

To delve into the genomic landscape of ST15 Kpn population in China, this study collected 287 genomes of clinical ST15 Kpn of China origin from the PATRIC database (Davis et al., 2020). Comparative genomic analyzes were performed to understand the spread of the ST15 Kpn strains across the country over 10 years. Then phylogenetic relationship, evolution, recent transmission, antimicrobial resistance and virulence gene profiling, pan-genome association, and plasmid content were screened.

## 2. Materials and methods

### 2.1. Genome collection and quality control

We retrieved all publicly available ST15 Kpn genome assemblies present in the PATRIC database on September 15, 2022 using the search terms “mlst = 15,” “genome status = WGS,” “host common name = Human,” “isolation country = China” and “genome quality = good.” These strains were all collected in China from 2012 to 2022. The corresponding metadata of the genomes was acquired from the PATRIC database and manually checked based on the NCBI Genbank database. Assembled genome and quality summary statistics were calculated with QUAST v5.2.0 and fastANI v1.33, respectively (Gurevich et al., 2013; Jain et al., 2018). All genomes passed the quality control with more than 99.5% of ANI and 85% of genome fraction with Kpn WSD411 (RefSeq: GCF_009884415.1) as an ST15 reference genome (Chen Y. et al., 2021). The MLST sequence type of each genome was also confirmed with Kleborate v2.2.0 (Lam et al., 2021).

### 2.2. Genome annotation

Genome annotation was performed using Prokka v1.13.4 (Seemann, 2014). Kleborate was used to identify the species identity, Kpn integrative conjugative element (ICEKp)-associated and plasmid-associated VF and AMR genes (Lam et al., 2021). Kleborate also assigns a virulence score and a resistance score for each genome. MOB-suite v3.0.3 was used to reconstruct plasmid content from each draft genome (Robertson and Nash, 2018). MOB-recon was used to analyze plasmid sequences, which includes MOB_typer to perform relaxase and replicon typing of plasmids, as well as generate MOB-cluster codes and host range information. The 28-bp fusion site was identified using the matchPattern function in the Biostrings R package with the specific sequence ‘AGATCCGNAANNNNNNNN TTNCGGATCT’ (Xu et al., 2021).

### 2.3. Phylogenetic and population structure analyzes

The core genome multi-alignment and SNP calling (cgSNP) was performed with Parsnp v1.2 from HarvestTools kit for the 287 genomes with collection dates and WSD411 as reference (Treangen et al., 2014). Pairwise SNP distances were calculated with SNP-sites v2.5.1 (Page et al., 2016). Phylogenetic trees were then constructed with RAxML v8.2.9 using the core genome SNP alignment after removing predicted recombination sites by Gubbins v2.1.0 (Stamatakis, 2014; Croucher et al., 2015). A general-time reversible nucleotide substitution model with a GAMMA correction for site variation was used for tree construction (bootstrap 1,000 with Lewis ascertainment correction). The output from Gubbins was loaded directly to BactDating v1.0.6, which accounts for branch-specific recombination rates, rather than simply ignoring recombinant regions (Didelot et al., 2014). Root-to-tip regression with a simultaneous inference of the best root location ( $R^{2}=0.39$ ) and tip-date-randomization performed within BactDating demonstrated a temporal signal in the data. 100 million Markov chain Monte Carlo (MCMC) steps were performed to generate a time-resolved tree using the mixed model for clock rate.

Phylogenetic clades were identified using fastBAPS, and a core-genome SNP (cgSNP) threshold of 16 was selected to define the putative transmission relationship, respectively (Tonkin-Hill et al., 2019; Zhang et al., 2022). Furthermore, a cgMLST allele calling was performed using chewBBACA suite with a public 2,358-gene cgMLST scheme for *K. pneumoniae*/var*iicola*/*quasipneumoniae* (Silva et al., 2018).^1^ Ancestral sequence reconstruction of each internal node of the phylogenetic tree was performed using the R package phangorn (Schliep, 2011). Terminal branch lengths were the number of substitutions mapped to each terminal branch. Both phylogenetic tree and metadata were visualized with R package ggtree (Yu et al., 2018). A median-joining haplotype network was reconstructed by PopArt v1.7 (Leigh and Bryant, 2015).

### 2.4. Pangenome construction and coincident analysis

A pangenome was generated from all genomes using Panaroo with default parameters, resulting in a gene absence-presence matrix (Tonkin-Hill et al., 2020). Pangenome sequences retrieved by Panaroo were annotated with eggnog-mapper v2.1.2 using eggnogDB v5.0.2 (Huerta-Cepas et al., 2019). The antibiotic resistance and virulence factor genes were screened using the Comprehensive Antibiotic Resistance Database (CARD) and the virulence factor database (VFDB), utilizing a protein identity threshold of 80% (Liu et al., 2022; Alcock et al., 2023). The absence-presence matrix of the accessory genome was plotted by the Uniform Manifold Approximation and Projection (UMAP) algorithm with R package umap (McInnes et al., 2018).

To determine if antibiotic resistance genes are co-circulating with each other accessory gene and each other, we adopted the program Coinfinder v1.2.0 (Whelan et al., 2020). Briefly, Coinfinder detects genes that associate or dissociate with other genes using a Bonferroni-corrected Binomial exact test statistic of the expected and observed rates of gene–gene association. We first ran Coinfinder on our combined dataset to identify all coincident associated gene pairs. Then we reran Coinfinder using the query flag to look specifically at simultaneously associated gene pairs involving KPC-2, OXA-232, and NDM-1, respectively. Gephi v0.9.4 was used to visualize a coincident gene network with the Fruchterman-Reingold layout algorithm (Bastian et al., 2009).

## 3. Results

### 3.1. Overview of sequenced ST15 Kpn clinical isolates in China

**Figure 1:**
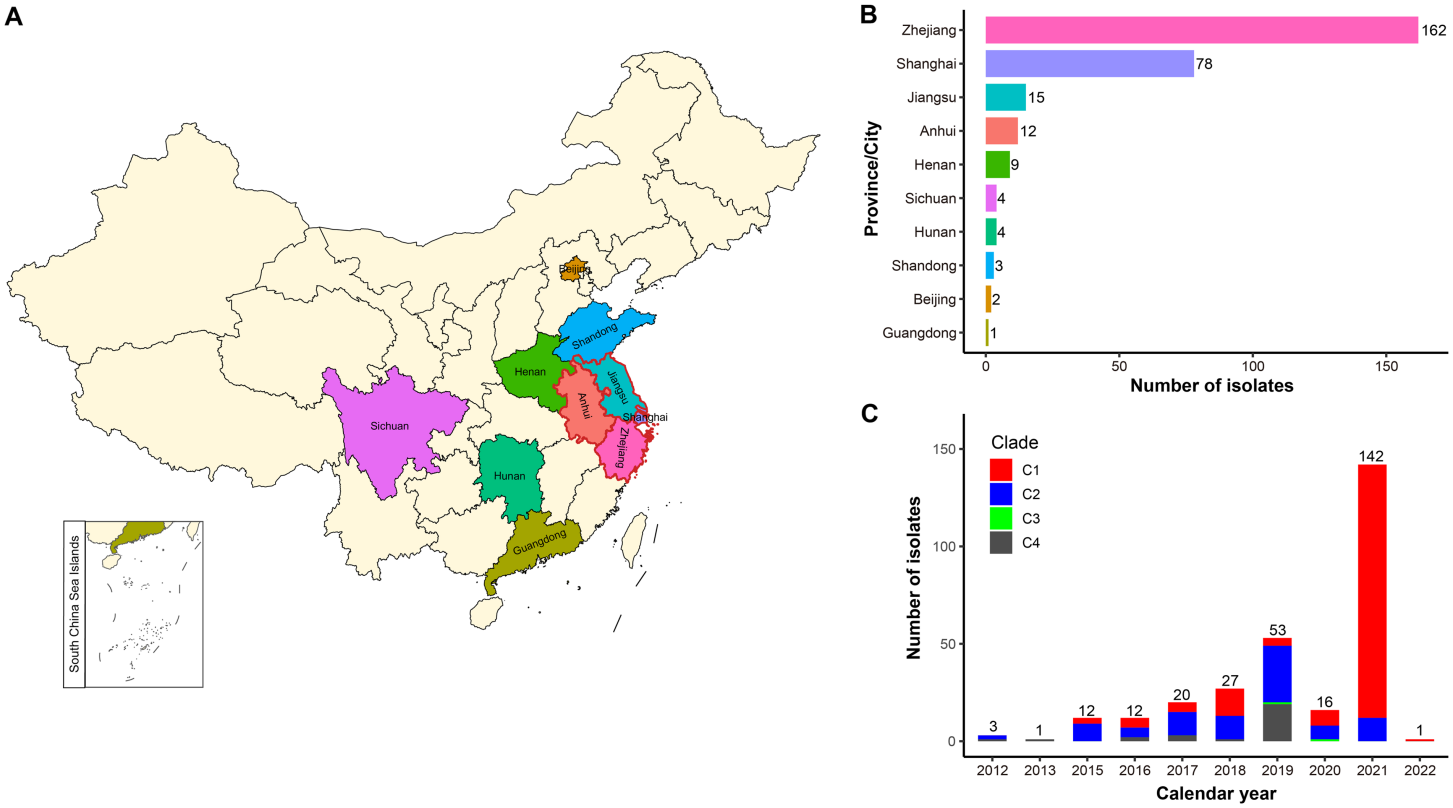
Geographic and temporal distribution of the studied ST15 Kpn strains in China. **(A)** The map of China shows the sampling areas for the enrolled strains, with the boundaries of the Yangtze River Delta (YRD) region highlighted in red. **(B)** Bar plot shows the prevalence of ST15 Kpn strains in 10 provinces and cities of China. **(C)** The stacked bar chart shows the frequency of ST15 strains sequenced in China each year from 2012 to January 2022 and the proportion of the four clades.

This study obtained 287 ST15 Kpn genome assemblies from China after database screening on September 15, 2022 (Supplementary Table S1). Zhejiang province has the most samples, accounting for 56.5% (162/287), followed by Shanghai city samples, accounting for 27.2% (78/287). Notably, the Yangtze River Delta (YRD) region in eastern China, encompassing Zhejiang, Jiangsu, and Anhui provinces and Shanghai city, was the top region infected with ST15 Kpn, accounting for 92.3% (265/287) of all samples (Figures 1A,B). The distribution of ST15 Kpn samples by year showed a rapidly growing tendency, except during the COVID-19 pandemic, which dominated 2020 (Figure 1C). Despite that, 72.1% (207/287) of the ST15 Kpn samples were isolated between 2019 and 2021.

### 3.2. Phylogenetic and genomic characteristics

**Figure 2:**
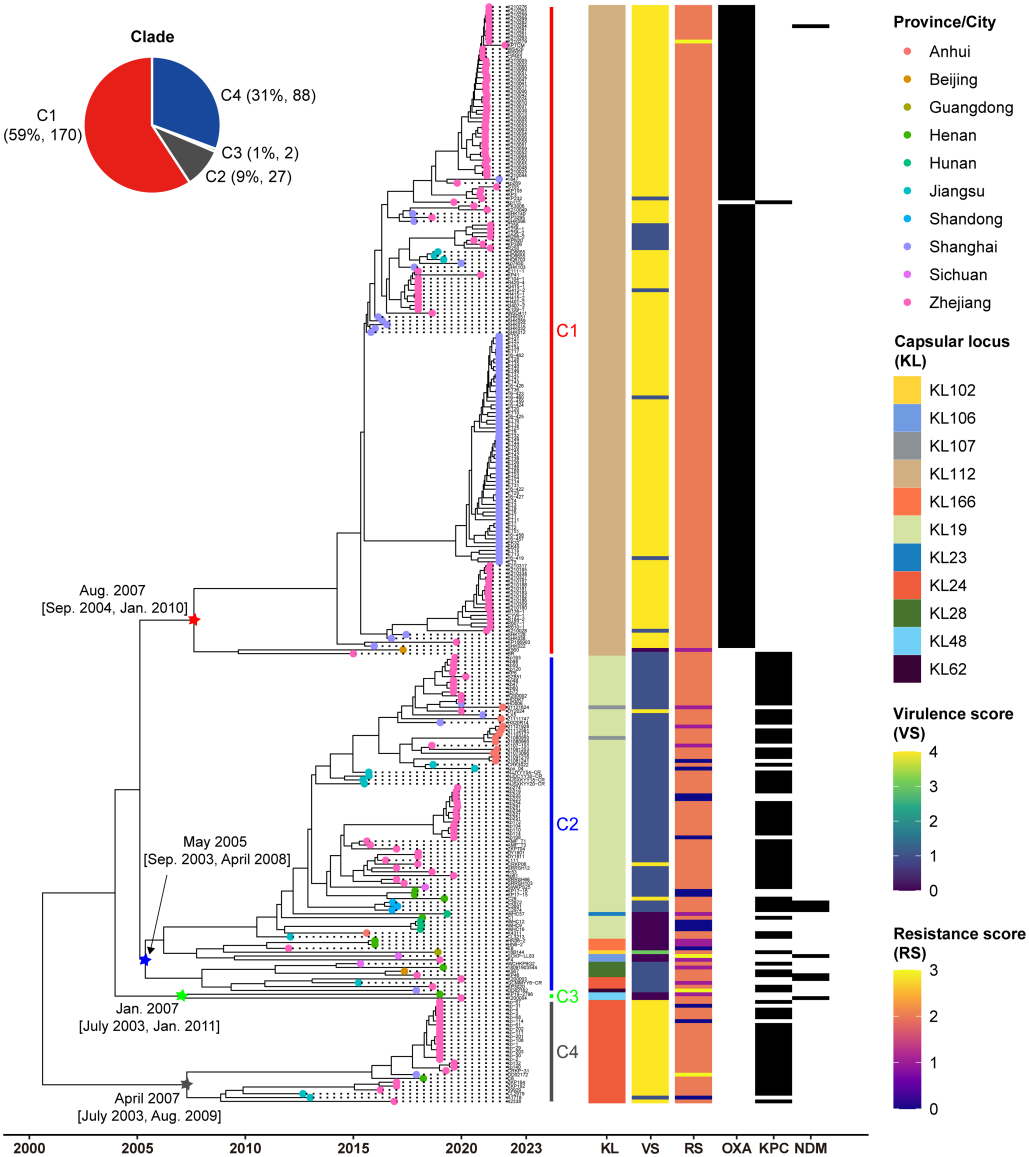
Low genetic diversity in China’s ST15 Kpn population. Dated phylogenomic tree of the core SNP analysis for China’s ST15 Kpn strains. The tree was constructed using BactDating and corrected for recombination using Gubbins. Individual nodes were colored by provinces as defined in the legend. The distinct clades, capsular locus (KL) type, virulence scores, and resistance scores of strains identified by Klebroate, presence of carbapenemase genes (OXA-232, KPC-2, and NDM) were shown on the tree (from inner to outer strips). The virulence score is based on the presence of *ybt*, *clb*, and *iuc* as follows: 0, none present; 1, *ybt* only; 2, *clb* without *iuc* (regardless of *ybt*; however, *ybt* is almost always present when *clb* is); 3, *iuc* only; 4, *iuc* and *ybt* without *clb*; and 5, all three genes present. Resistance scores are calculated as follows: 0 = no ESBL or carbapenemase, 1 = ESBL without carbapenemase (regardless of colistin resistance); 2 = carbapenemase without colistin resistance (regardless of ESBL); 3 = carbapenemase with colistin resistance (regardless of ESBL). The estimated origin times and 95% CI of four clades are shown at the relevant nodes. The time scale is indicated at bottom.

All ST15 Kpn strains were closely related with a maximum pairwise SNP distance of 222 SNPs, raising the possibility of clonal expansion of a common strain. The most-recent common ancestor of the 287 strains with isolation dates was estimated to emerge in August 2000 (95% confidence interval, April 1996 to October 2003) (Figure 2). The population of ST15 Kpn in China was further divided into four monophyletic clades based on a cgSNP/fastBAPS analysis that were C1, C2, C3, and C4 from top to bottom on the tree. There were 170 (59.2%) strains in C1, 88 (30.7%) in C2, 2 (0.7%) in C3, and 27 (9.4%) in C4, respectively. We inferred that C2 emerged first in May 2005, while the other three clusters emerged in the same year, 2007. Moreover, the latest sampling times for C1 and C2 are January 12, 2022, and December 1, 2021, respectively. In contrast, C3 and C4 have no new isolates after 2020 and 2019, respectively.

The capsule type (KL), VF and AMR characteristics revealed by Kleborate were further mapped on the phylogeny (Figure 2). A total of 11 distinct KL types were identified, with KL112 (59.2%, 170/287), KL19 (24.7%, 71/287), and KL24 (10.5%, 30/287) emerging as the predominant ones (Supplementary Figure S1). Significantly, unique capsule types, namely KL112, KL48, and KL24, corresponded to C1, C3, and C4, respectively. C2 displayed a diversity of nine KL types, with KL19 as the predominant one, accounting for 80.7% (71/88), and included three KL24 strains.

C1 and C4 displayed significantly higher virulence than C2 and C3 due to their aerobactin and yersiniabactin VFs (Fisher’s exact test *p* < 0.001). Among the studied strains, 91.6% (263/287) were CP-Kpn, of which 33.1% (88/263) producing KPC-2, 57.6% (167/263) producing OXA-232, 1.1% (3/263) producing NDM, 1.5% (4/263) producing both KPC-2 and NDM, and 0.4% (1/263) producing both OXA-232 and NDM. Besides, 64.1% (184/287) of the dataset presented *iucABCD* and *rmpA2* genes, and from this 98.9% (182/184) were also carbapenemase producer. These MDR-hvKpn events included 156 C1, 2 C2 and 24 C4 strains. Associations were observed between carbapenemases and clades, with significant enrichment of OXA-232 in C1 and KPC-2 in both C2 and C4 (Fisher’s exact test *p* < 0.001). Drug resistance also significantly varied among different clades (Fisher’s exact test *p* < 0.001). We also found four strains DD02162 (C2), DD02172 (C4), K210279 (C1), and SCKP-LL83 (C2) exhibited the highest resistance scores of 3 for both carbapenemases and colistin resistance. Colistin resistance mechanisms included the presence of the colistin-resistant *mcr-1* gene in SCKP-LL83 and inactivated mutations in the *mgrB* gene in the other three strains.

To assess whether the genotypes of ST15 Kpn strains in China differed from those of strains isolated from other global areas, we further included the genomic data of 293 strains collected from other parts of the world (Supplementary Table S2) (Rodrigues et al., 2023). Similar to the strains in China, the most common KL type in these global strains was KL112 (49.1%, 144/293), but the proportion of KL24 (35.2%, 103/293) was higher than KL19 (5.1%, 15/293). The maximum-likelihood phylogenetic tree of all 580 strains showed that C1-C4 in China all had highly homologous strains from other global regions, especially Asia and Europe (Supplementary Figure S2). Except for few strains in C1 and C2, most of the strains collected from China were monophyletic in the phylogenetic tree.

### 3.3. Clonal transmission evidence in China’s ST15 Kpn isolates

**Figure 3:**
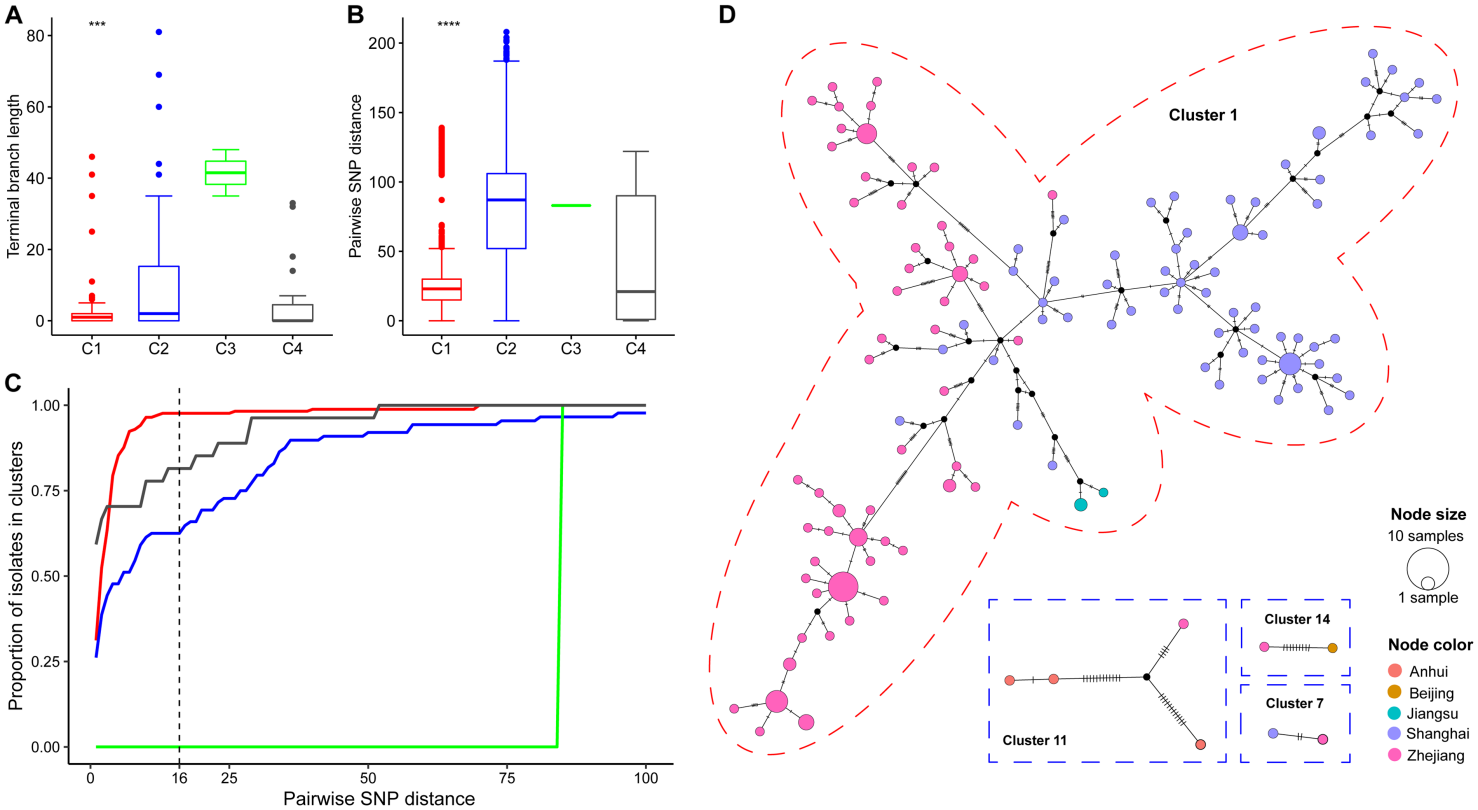
Genomic epidemiology of China’s ST15 Kpn. **(A)** The distribution of terminal branch lengths for different clades (colored as in Figure 2). **(B)** The distribution of pairwise SNP distances for different clades. Three and four asterisks indicate Wilcoxon test *p*-values smaller than 0.001 and 1e-04, respectively. **(C)** Proportion of isolates from each clade that belong to clusters (y axis) defined at different thresholds for maximum pairwise SNP distances (x axis). **(D)** Median-Joining network generated for four cross-regional transmission clusters using PopArt. Clusters are outlined with a dashed circle representing the color of the clade in which their strain belongs. A circle represents a haplotype. The circle area represents the frequency of strains, and hatch marks across branches indicate mutational steps on the edges. Black dots indicate inferred missing isolates. The color inside each circle represented the isolation area.

The diversity metrics of their subtrees were calculated to assess the difference in transmissibility and capability of causing active disease in infected hosts among the four clades. The C1 phylogeny had significantly shorter terminal branch lengths than the other clades (Figure 3A, Wilcoxon test *p* < 0.001). Strains belonging to C1 were genetically more similar than those belonging to different clades, as indicated by the smaller median pairwise SNP distance (Figure 3B, Wilcoxon test *p* < 0.001). Furthermore, we explored the distribution of potential transmission clusters using a range spanning 1 ~ 100 SNPs of maximum pairwise SNP distance thresholds to define a transmission cluster (Figure 3C). Notably, the proportion of strains belonging to transmission clusters was significantly higher among C1 strains than in other clades.

The genomic distance matrices computed on the cgMLST concatenate and cgSNP alignment was significantly correlated (Mantel test, *p* < 0.001; Spearman test R = 0.81, *p* < 0.001). According to a recently published molecular epidemiology study of CP-Kpn in Shanghai, the clonal clusters were defined using a cutoff of 16 SNPs (Zhang et al., 2022). Here, 245 (85.4%) of the 287 strains were detected in 18 clonal clusters, ranging in size from two to 159 (Supplementary Figure S3). Furthermore, a cgMLST typing analysis retrieved a similar clustering result that 247 (86.7%) of the 287 strains into 15 clonal clusters at an allele distance threshold of ten, which has been used to correctly group all surgical intensive care unit outbreak strains in a hospital in Beijing (Zhou et al., 2017).

The cgSNP clustering rate of C1 (97.6%, 166/170) was significantly higher than that of C2 (64.8%, 57/88), C3 (0, 0/2), and C4 (81.5%, 22/27) (chi-square test, *p* < 0.001). In addition, 77.8% (14/18) of clonal clusters involved transmissions that occurred within 1 year. The longest for the other four clonal clusters is 6.2 years, followed by 3.0, 1.9, and 1.7 years. Notably, there were five cgSNP-based clonal clusters including 167 strains involving recent transmissions across different provinces (Figure 3D). Four clusters (cluster 1, 7, 11 and 14) were located in the YRD region, and only one cluster (cluster 14) was the transmission between Beijing and Zhejiang. The largest cluster (cluster 1), including 93.5% (159/170) strains of C1, isolated from 2015 to 2022, revealed a large-scale continuous spreading of the ST15 OXA-232-CP-Kpn of C1 in the YRD region.

### 3.4. Plasmids profiling based on the complete genomes

**Figure 4:**
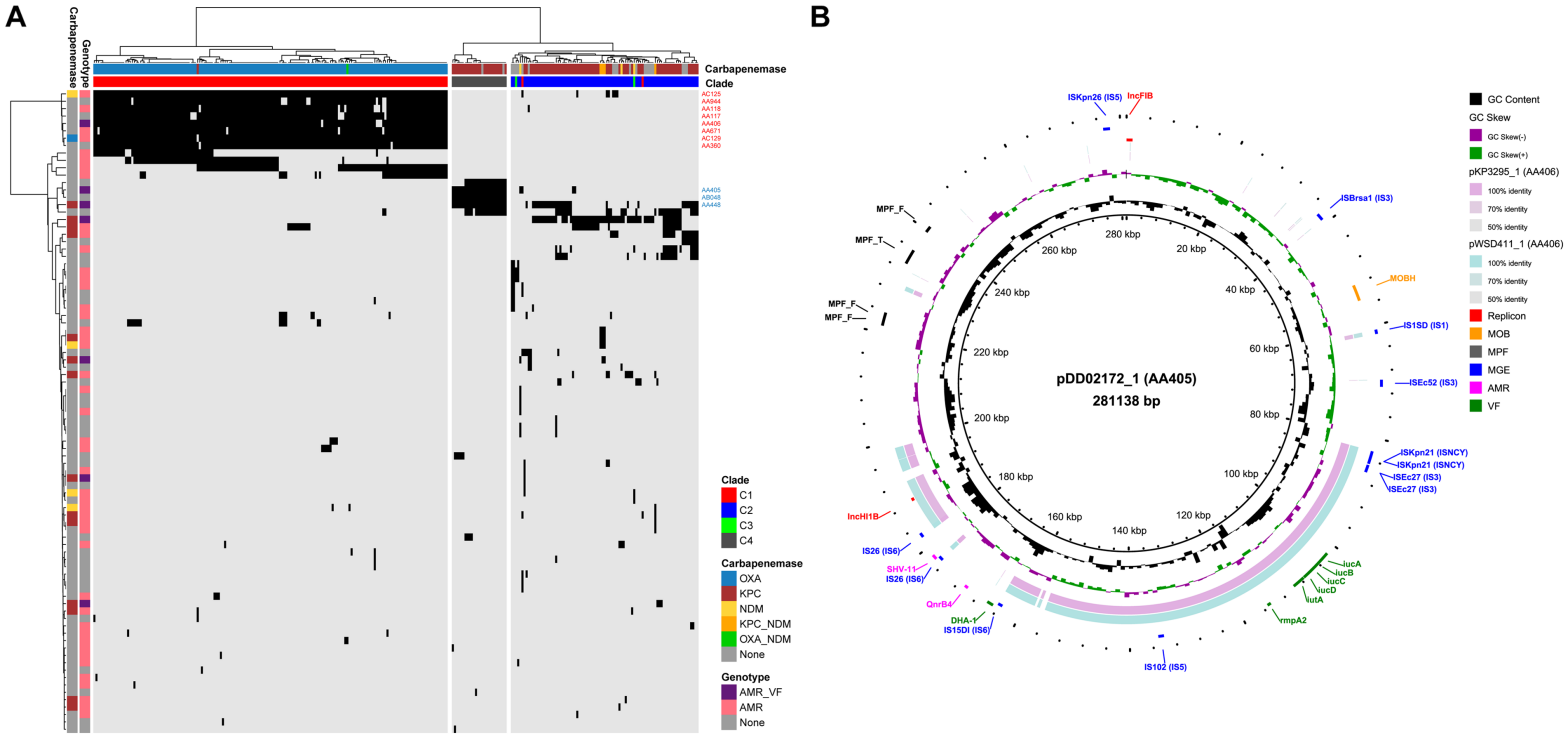
Plasmid profiling of China’s ST15 Kpn. **(A)** Heat map of hierarchical clustering based on the presence (black) and absence (light gray) of 88 PCs (row) among 287 strains (column). The colors on the top of the heat map represent the clade to which the strain belongs and the carbapenemase it encodes. The colors on the left side of the heat map indicate whether each PC encodes AMR and/or VF genes and the carbapenemase it encodes. The 8 CC-PCs in C1 and 3 CC-PCs in C4 are marked. **(B)** Alignment of the complete plasmids of AA405 and AA406 using BLAST Ring Image Generator (BRIG) (Alikhan et al., 2011). The plasmid pDD02172_1 in AA405 is used as the reference. The outer colored labels refer to the annotation of replicon, MOB, mating pair formation (MPF), mobile genetic element (MGE), AMR and VF gene, respectively.

To reveal the plasmid communities shared among China’s ST15 Kpn population, we adopted three tools in MOB-suite to all genomes: MOB-recon for plasmid sequence identification, MOB-typer for plasmid typing, MOB-cluster for plasmid clustering, respectively. A total of 2,101 plasmids were detected and grouped into 88 plasmid clusters (PCs) at a mash distance threshold of 0.05 (Supplementary Table S3A). Only 31 of these PCs contained more than 5 plasmids, indicating the complexity of plasmid content. An average number of PCs per genome was 7.4 (between 1 and 12), and the number increased from C3 (average = 3) to C2 (average = 3.6) to C1 (average = 9.7) to C4 (average = 5.0), with a statistically significant difference (ANOVA test, *p* < 0.001) (Figure 4A). The hierarchical clustering based on the presence and absence of PCs among all strains showed a clear separation of the plasmid content between C1, C2 and C4, as well as between KPC-2 and OXA-232. Furthermore, since there was no common PC present in all strains, we focused on the core PC within each clade (CC-PC), that was, the PC that appear in more than 80% of the members. We found that C1 and C4 had 8 and 3 CC-PCs, respectively, while C2 and C3 had none. Based on the 16 complete genomes, we found that 4 CC-PCs belong to 3 Inc. replicon families (FIB, HI1B and U), 4 CC-PCs belonged to 2 Col replicon types. Besides, 2 CC-PCs were conjugative, 4 CC-PCs were mobilizable, and 5 CC-PCs were non-mobilizable.

We identified 60.2% (53/88) of the PCs carrying AMR genes, with an average of 2.8 AMR genes per PC (ranging from 1 to 17) (Supplementary Table S3B). KPC-2 was found in 14 PCs including a CC-PC (AA448, IncU-type), 12 of which were conjugative or mobilizable. NDM was present in four PCs including a CC-PC (AC125, IncFIB-type), two of which were conjugative or mobilizable, while OXA-232 was exclusively detected in a mobilizable CC-PC (AC129, rep_cluster_1195). Moreover, we found the colistin resistance gene *mcr-1* in two PCs within C2: AA378, which carried one AMR gene, and AA738, which encoded 17 AMR genes. Notably, seven of these AMR PCs also carried VF genes (Supplementary Table S3C). Among them, the VF genes *iucABCD* and *rmpA2* coexisted on 183 plasmids, forming two F-type PCs: AA405 in C4 (KPC-2-producing) and AA406 in C1 (OXA-232-producing). Sequence alignment of two representative plasmids, pDD02172_1 (AA405) and pWSD411_1 (AA406), revealed their homology with a mean identity of 86.9% and coverage of 44.3% (pDD02172_1 as the reference) (Figure 4B). There were IS sequences belonging to the IS*NCY*, IS*3* and IS*6* families at both ends of the homologous region containing the VFs. However, AA405 was conjugative, while AA406 lacked both relaxase and mate-pair formation, making it non-mobilizable. Interestingly, we detected the non-mobilizable pWSD411_1 has the potential to co-transfer with a conjugative F-type plasmid pWSD411_2 for both sharing the 28-bp fusion site (Xu et al., 2021).

### 3.5. An open structure of China’s ST15 Kpn pan-genome

**Figure 5:**
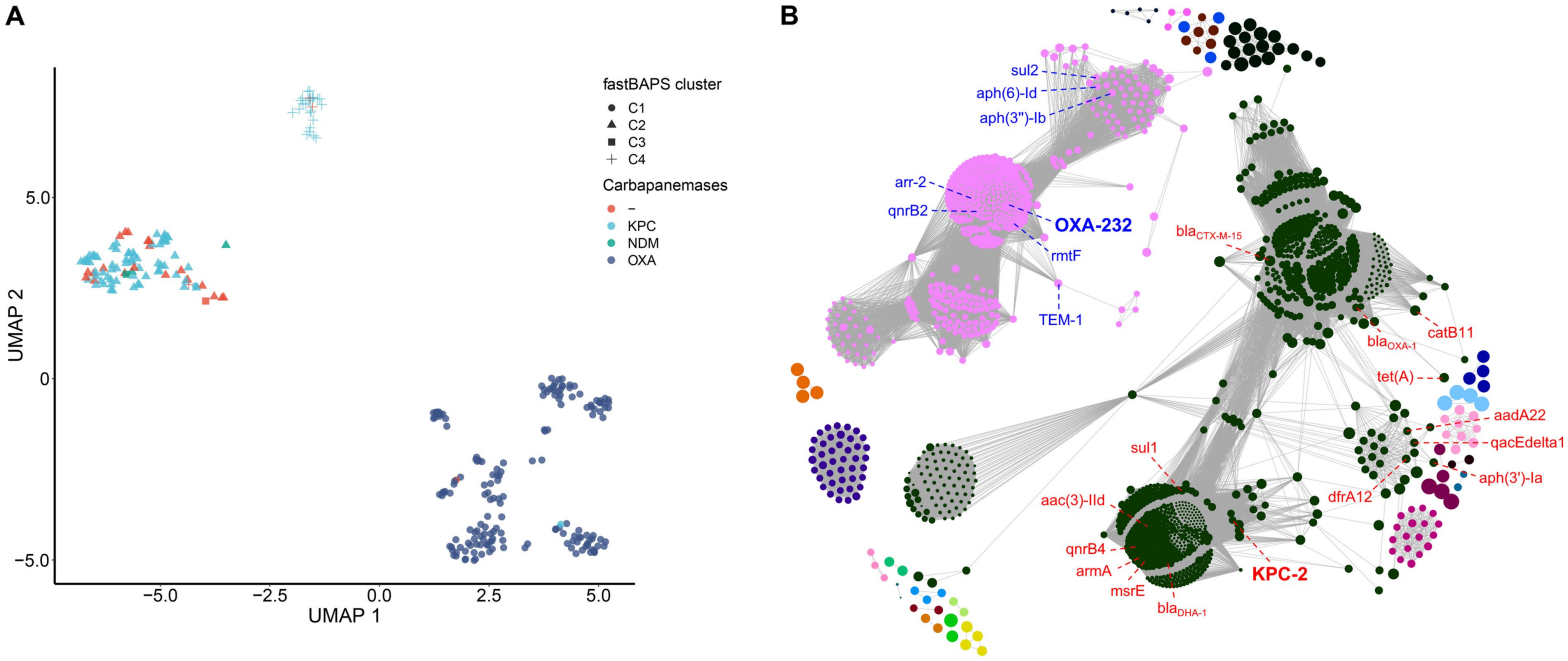
Pan-genome modeling of China’s ST15 Kpn population. **(A)** Visualization of the UMAP two-dimensional representation of the pan-genome. Strains in different clades were shown with different shapes and colored by different carbapenemase genes. **(B)** Network diagram created with Gephi using output from Coinfinder carried on China’s ST15 Kpn pan-genome. Nodes are colored by connected components (coincident gene sets). The size of a node is proportional to the gene’s D value (Whelan et al., 2020).

To characterize the genomic diversity of the analyzed 287 ST15 Kpn genomes, a pan-genome was constructed. This pan-genome consists of 4,539 core and 4,377 accessory genes. The simulated gene accumulation curves showed that the numbers of the core genes decreased continually with the addition of new strains, as expected when sampling more diverging genomes of a species (Supplementary Figure S4). Heap’s law modeling ( $n=\kappa N^{\gamma }$ ) of the gene presence-absence revealed a γ value of 0.1 less than 1, demonstrating the open state of the pan-genome. Displaying the genomes using a UMAP approach directly on the absence-presence of accessory genes showed a clear separation of the three main clades (C1, C2, and C4) identified by fastBAPS based on the cgSNP (Figure 5A). Although C2 is closer to C1 in terms of genetic distance, the accessory genome composition of C2 is more comparable to that of C4. This phenomenon suggested that the composition of accessory genes between different strain clusters may be related to the mechanisms of carbapenem resistance.

### 3.6. Coincident genes associated with carbapenem-resistance genes

To further explore the coincident gene relationships within the pan-genome, a gene co-occurrence network was inferred by Coinfinder (Figure 5B). It contained a total of 228,491 significant gene-to-gene relationships, including 1,691 coincident genes, accounting for 38.6% (1,691/4377) of all accessory genes (Bonferroni-corrected binomial exact test, *p* < 0.05). These associated gene pairs were further clustered into 25 associate components. There were two large components in the network, containing 1,037 and 508 genes, respectively, and occupied 91.4% (1,545/1691) of all coincident genes. The first component included the KPC-2 with other 15 AMR genes [*bla_CTX-M-15_*, *catB11*, *tet(A)*, *dfrA12*, *aph(3″)-Ia*, *sul1*, *aadA22*, *qacEdelta1*, *bla_OXA-1_*, *msrE*, *mphE*, *armA*, *qnrB4*, *bla_DHA-1_* and *aac(3)-IId*] contributed to the resistance to cephalosporin, penam, tetracycline, diaminopyrimidine, aminoglycoside, sulfonamide, phenicol, streptogramin, macrolide, fluoroquinolone and cephamycin antibiotics. The second component included the OXA-232 with other seven AMR genes [*TEM-1*, *sul2*, *aph(6)-Id*, *aph(3″)-Ib*, *arr-2*, *qnrB2* and *rmtF*] contributed to the resistance to penem, cephalosporin, monobactam, sulfonamide, aminoglycoside, rifamycin and fluoroquinolone antibiotics. All other components were far smaller, ranging from 2 to 42 genes. Two other gene clusters also included antibiotic resistance genes; one of 9 genes included *qnrS1*, and one of 42 had *ramR*.

When we only examined coincident gene–gene relationships involving the three carbapenemases, including KPC-2, OXA-232, and NDM-1, we identified 383, 391, and 0 coincident genes, respectively. Six genes directly coincident with KPC-2 were AMR genes: *sul1*, *mphE*, *DHA-1*, *qnrB4*, *armA,* and *msrE*. The genes directly coincident with OXA-232 included three AMR genes (*rmtF*, *qnrB2,* and *arr-2*), and two VF genes (*iucA* and *rmpA*), related to aerobactin and mucoid phenotype A regulation, respectively. In addition, we found that all the AMR and VF genes coincident with OXA-232 and KPC-2 were located on the plasmid according to the complete genomes WSD411, DD02162, and KP46 (Supplementary Tables S2B,C).

By comparing the functions between the KPC-2’s and OXA-232’s coincident genes, we found that genes related to post-translational modification, protein turnover, and chaperones (COG category code O) and intracellular trafficking, secretion, and vesicular transport (COG category code U) were much more prevalent in the KPC-2’s coincident genes (Supplementary Figure S5A). In contrast, genes related to replication, recombination, and repair (COG category code L), inorganic ion transport and metabolism (COG category code P), and signal transduction mechanisms (COG category code T) were more likely to be included in the OXA-232’s coincident genes. Furthermore, 71.5% (274/383) of the KPC-2’s coincident genes and 80.1% (313/391) of the OXA-232’s coincident genes were plasmid-mediated (Supplementary Figure S5B). There was no significant difference in the distribution of KPC-2’s and OXA-232’s coincident genes on chromosome and plasmid (chi-square test, *p* = 0.402).

## 4. Discussion

In this study, by screening all public ST15 Kpn genomes, we found that the isolation frequency of ST15 Kpn in China has continued to increase over the past decade. The ST15 Kpn in China originated in 2000 and has differentiated into four distinct clades. The origin of these clades was as early as 2005 and as late as 2008, and some strains are still emerging in 2022. The predominant KL types in the studied strains are KL112, KL19, and KL24. A comparison with strains from other global regions indicates similarities in KL types, with KL112 being the most common (Rodrigues et al., 2023). This suggests a global distribution of certain KL types within ST15 Kpn population. Notably, most new cases occurred in the YRD region, eastern China, between 2019 and 2021. With the high resolution provided by genomics, we revealed that up to 85% of isolates were due to recent transmission. C1 and C4 displayed higher virulence, likely contributing to the severity of infections they cause. Recently, numerous nosocomial outbreaks of ST15 Kpn were reported in the YRD region including Hangzhou, Lishui, Wenzhou, Yancheng, Jiaxing and Shanghai (Li et al., 2019; Jia et al., 2021; Zhu Z. et al., 2021; Huang et al., 2022; Wu et al., 2023; Zhang et al., 2023). The YRD region is one of the most economically active regions in China and attracts a large number of migrant workers from Yunnan, Sichuan, and Anhui provinces every year. This finding suggested that there was a high level of transmission of ST15 CP-Kpn between hospitals by patient transfer. However, the transmission of CP-Kpn within and between hospitals remains largely unexplored in China (Cienfuegos-Gallet et al., 2022). Meanwhile, it should be pointed out that the YRD region has more medical resources than other central and western regions in China, which may be one of the reasons why most of the currently sequenced strains originate from this region. Accordingly, we speculate that ST15 Kpn has been widely disseminated in China in recent years.

The emergence and expansion of CP-Kpn have resulted in a bottleneck in effective antimicrobial treatment (Zong et al., 2021). Worryingly, through AMR gene testing, we found that 92% of the enrolled ST15 Kpn strains are CP-Kpn, and 69% of which are MDR-hvKpn with *iuc* and *rmpA2*. MDR and hv are typically observed in separate Kpn populations. However, convergent strains with both properties have been documented and potentially pose a high risk to public health in the form of invasive infections with limited treatment options (Arcari and Carattoli, 2023). OXA-232 is the most detected carbapenemase in China’s ST15 CP-Kpn, followed by KPC-2, while NDM is relatively rare. Notably, the co-occurrence of NDM with both KPC-2 and OXA-232 has already appeared. Therefore, the emergence of NDM has become a growing public health threat and represents a new challenge for the treatment of infectious diseases (Gao et al., 2020).

Notably, our pan-genome analysis provides valuable insights into the relationship between genomic diversity, clade-specific differentiation, and the presence of carbapenem resistance genes among ST15 Kpn. First, phylogenetic analysis based on the cgSNP showed the emergence of distinct clades (C1, C2, C3, and C4) is associated with the presence of these carbapenemase genes. Second, the observed open pan-genome structure reflects the remarkable diversity within ST15 Kpn and indicates that they can exchange genetic material (Holt et al., 2015). The distinct clades exhibit varying accessory gene profiles, and most of the accessory genes are coincides with KPC-2 or OXA-232 and located on the plasmids. Therefore, the pan-genome of China’s ST15 CP-Kpn has already differentiated into KPC-2-type and OXA-232-type structures at both core and accessory genomes. In addition, these coincident accessory genes include some AMR genes confer to aminoglycoside, sulfonamide, cephalosporin and fluoroquinolone, and aerobactin and regulation VF genes. Indeed, fluoroquinolone resistance appears to confer a fitness advantage to high-risk clones of various species, particularly among the elderly and individuals with prolonged healthcare center exposure, which is a known risk factor for acquiring additional antibiotic resistance genes (Redgrave et al., 2014; Fuzi et al., 2020). These evidence suggested an adaptive evolution of plasmid-mediated large-scale horizontal gene transfer among China’s ST15 Kpn strains.

We found 88 different PCs in China’s ST15 Kpn strains and high variation in plasmid content among different clades. C1 and C4 display a more stable and clade-specific plasmid repertoire with a higher number of CC-PCs. In contrast, C2 and C3 lack CC-PCs, indicating a less stable plasmid composition. The overrepresentation of F and Col plasmids and high heterogeneity of small plasmids in China’s ST15 Kpn was also similar to a recent global ST15 Kpn research (Rodrigues et al., 2023). Notably, up to 60% of the PCs in our study encoded at least one AMR gene, with a maximum of 17 AMR genes, and 7 of them also carried VFs. Comparing KPC-2 and NDM, there is only 1 PC carrying OXA-232, suggesting that the spread of the KPC-2 and NDM is more complex than that of OXA-232 in China’s ST15 Kpn (Zhang et al., 2023). There is one mobilizable CC-PC encoding OXA-232 and one conjugative CC-PC encoding KPC-2 in C1 and C4, respectively.

We emphasize that the *iucABCD* and *rmpA2* genes in all MDR-hvKpn genomes are located on plasmids. Although these plasmids belong to two PCs, one was conjugative and the other could co-transfer with a conjugative F-type plasmid in the same genome (Xu et al., 2021). They have a certain degree of homology and might be formed through recombination mediated by IS sequences (Acman et al., 2022). The presence of both VF and AMR genes, especially the carbapenemase genes, and *iuc* and *rmpA2* VFs on plasmids enables simultaneous transfer in a single event and potentially rapid emergence of MDR-hvKpn clone (Tang et al., 2020). Moreover, the presence of colistin resistance genes on specific plasmids in C2 strains is a concerning development, as colistin is often considered a last-resort antibiotic (Zong et al., 2021).

We acknowledge the imperfect nature of the ST15 Kpn dataset we used. First, only the PATRIC database was used for sample screening, which is largely biased and commonly not well-curated. There was insufficient diversity among China’s ST15 strains included in the study. Second, the available metadata can significantly impact the dating estimation and may not be correct. Third, there was no related experiment to demonstrate both drug resistance and virulence potential from genomic detection.

In conclusion, this study provides a comprehensive view of the molecular epidemiology and genetic diversity in the China’s ST15 Kpn population. Our findings demonstrated that clonal transmission was the leading cause of the increasing incidence of infections due to the ST15 CP-Kpn during the past 5 years. The variety of the cgSNP-based phylogeny, the composition of accessory genes, and the plasmid profiles correlated to the two different carbapenem genes, OXA-232 and KPC-2. These findings provide essential perspectives into ST15 CP-Kpn and highlight the urgent need for medical institutions to strengthen surveillance to prevent these novel strains from further disseminating in hospital settings and the community.

## Data availability statement

The datasets presented in this study can be found in online repositories. The names of the repository/repositories and accession number(s) can be found in the article/Supplementary material.

## Author contributions

LF: Conceptualization, Data curation, Formal analysis, Investigation, Writing – original draft, Writing – review & editing. MZ: Data curation, Formal analysis, Investigation, Writing – original draft, Writing – review & editing. ZF: Data curation, Formal analysis, Writing – original draft.

## Footnotes

^1^ https://www.cgmlst.org/ncs/schema/2187931/

## Supplementary material

Supplementary Table S1 Metadata and genotyping results of 287 public China’s ST15 Kpn genomes from the PATRIC database.

Supplementary Table S2 Metadata of 293 public ST15 Kpn genomes worldwide.

Supplementary Table S3 Plasmid-typing **(A)** results from the MOB-suite software and AMR **(B)** and VF **(C)** genes content of each plasmid.

Supplementary Figure S1 Statistics of all serotypes.

Supplementary Figure S2 Phylogenetic tree of 287 China’s and 293 global ST15 Kpn strains.

Supplementary Figure S3 Statistics of members of all transmission clusters based on a threshold of 16 SNPs.

Supplementary Figure S4 Simulations of the increase of the pan-genome size and the decrease of core-genome size.

Supplementary Figure S5 COG category annotation **(A)** and genomic localization **(B)** statistics of the KPC-2’s and OXA-232’s coincident gene.
