# Supplementary figures and images for: Population genomic analysis of clinical ST15 Klebsiella pneumoniae strains in China
Source: Front Microbiol. 2023 Nov 15;14:1272173. doi: 10.3389/fmicb.2023.1272173 (PMC10684719; doi:10.3389/fmicb.2023.1272173)

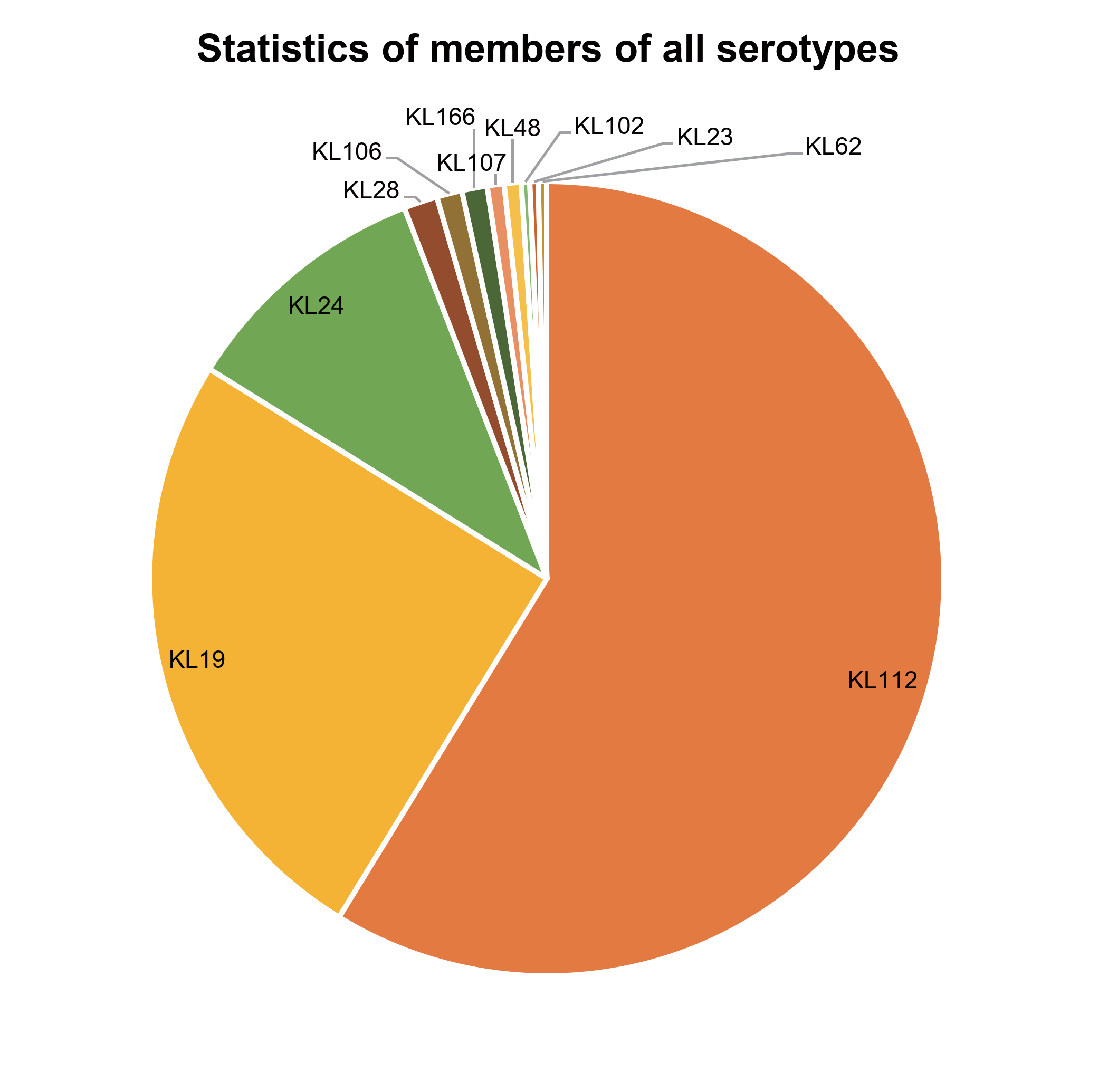

Supplement: Supplementary Figure S1 — Statistics of all serotypes. [file Image_1.TIF]

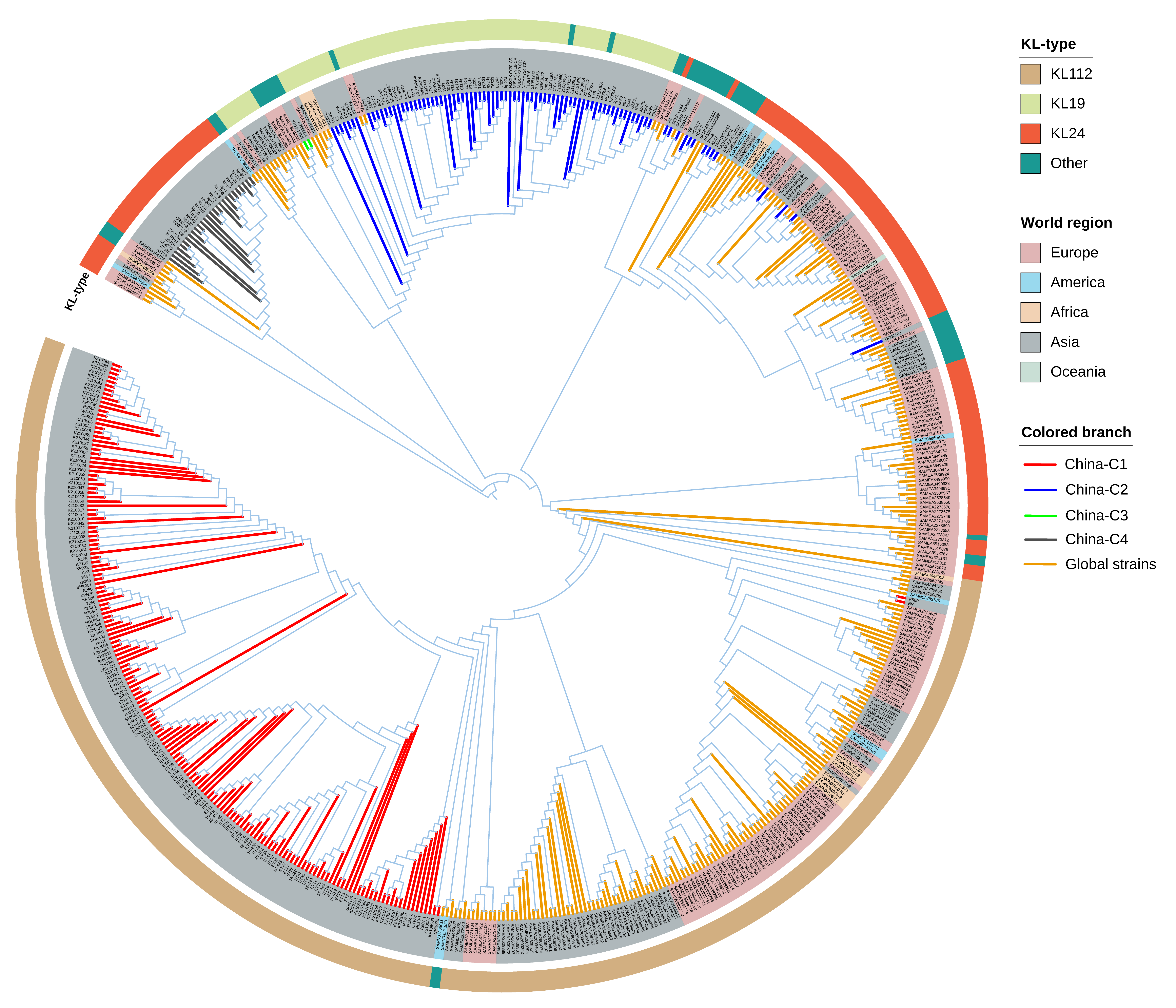

Supplement: Supplementary Figure S2 — Phylogenetic tree of 287 China’s and 293 global ST15 Kpn strains. [file Image_2.TIF]

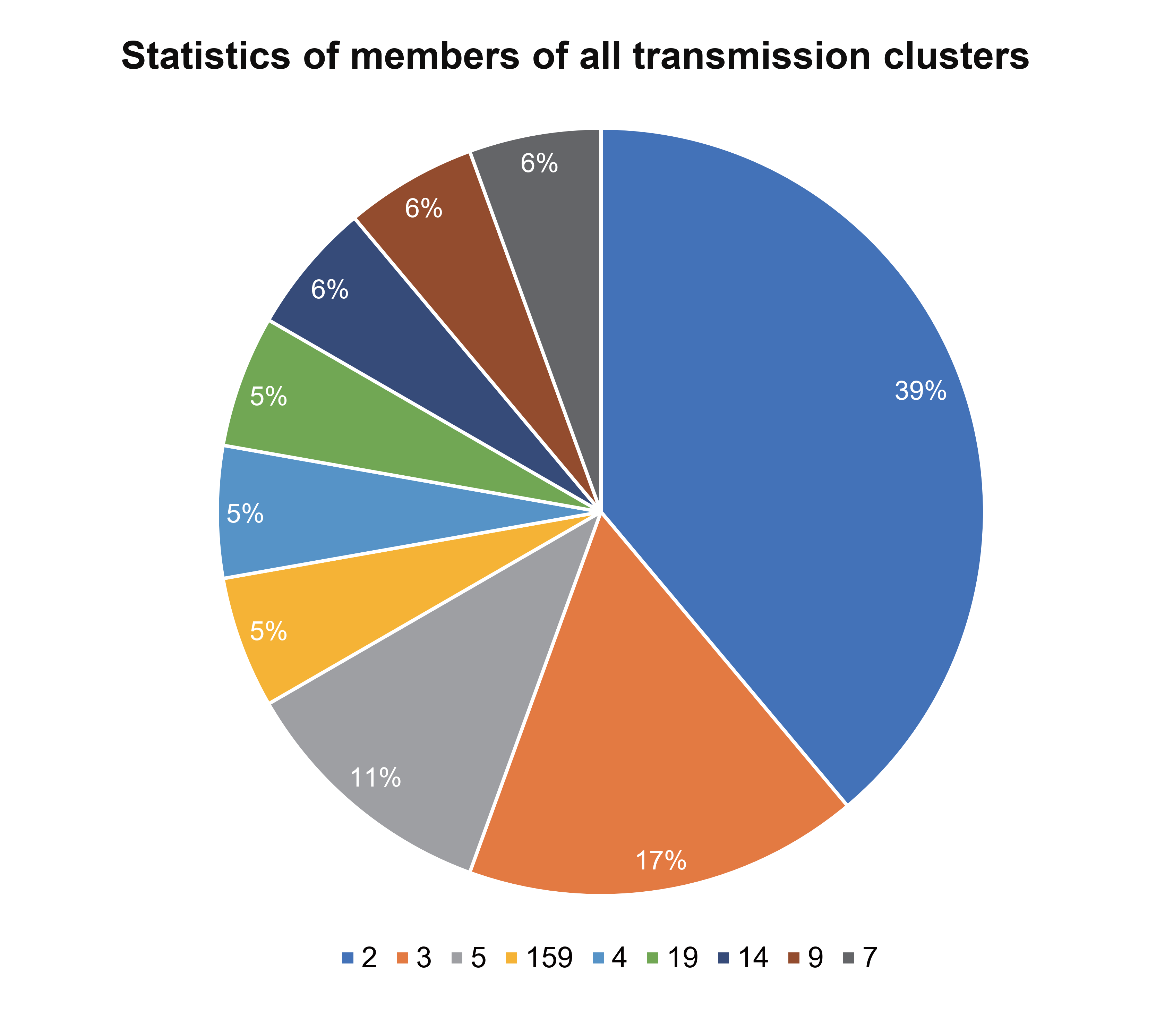

Supplement: Supplementary Figure S3 — Statistics of members of all transmission clusters based on a threshold of 16 SNPs. [file Image_3.TIF]

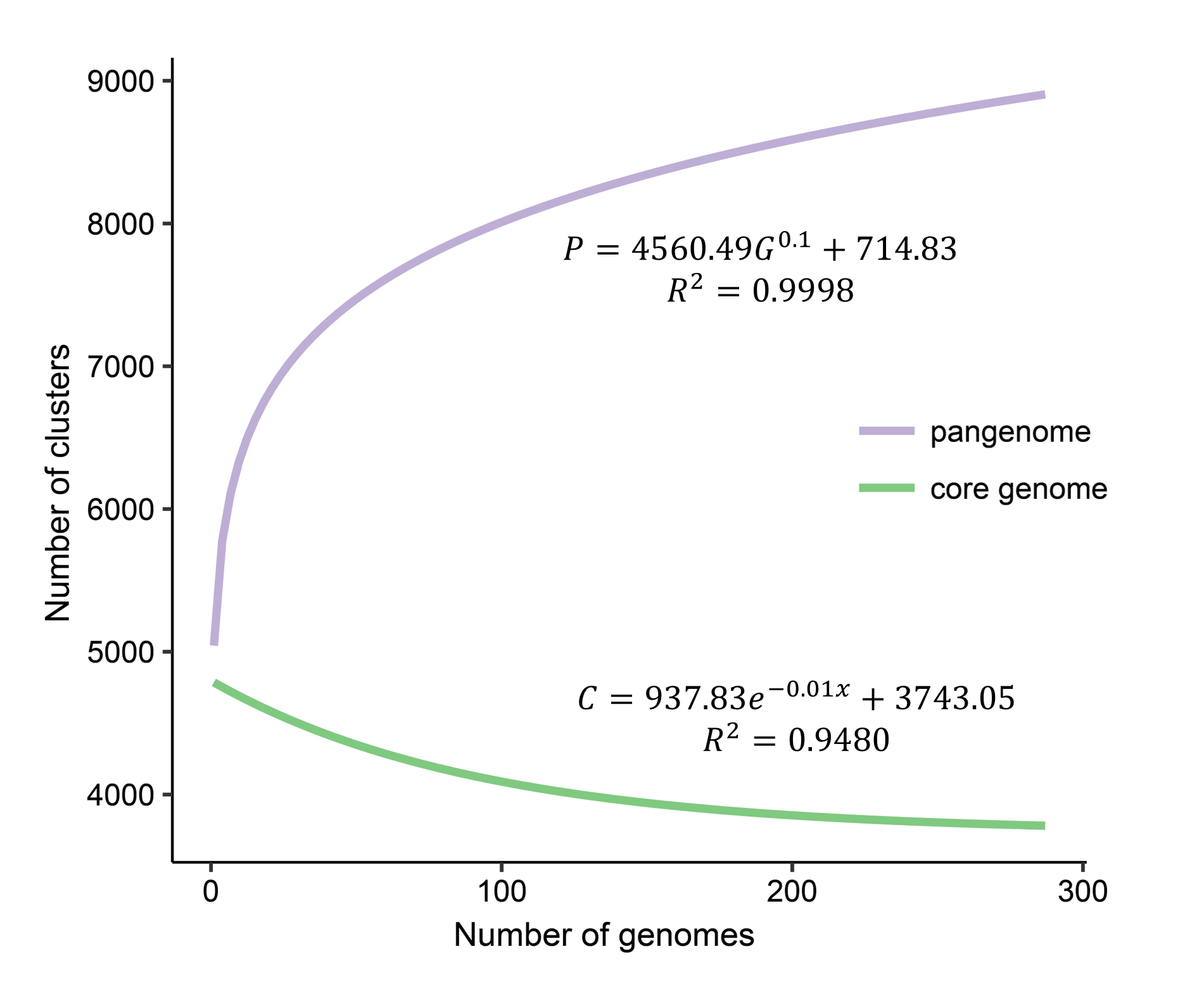

Supplement: Supplementary Figure S4 — Simulations of the increase of the pan-genome size and the decrease of core-genome size. [file Image_4.TIF]

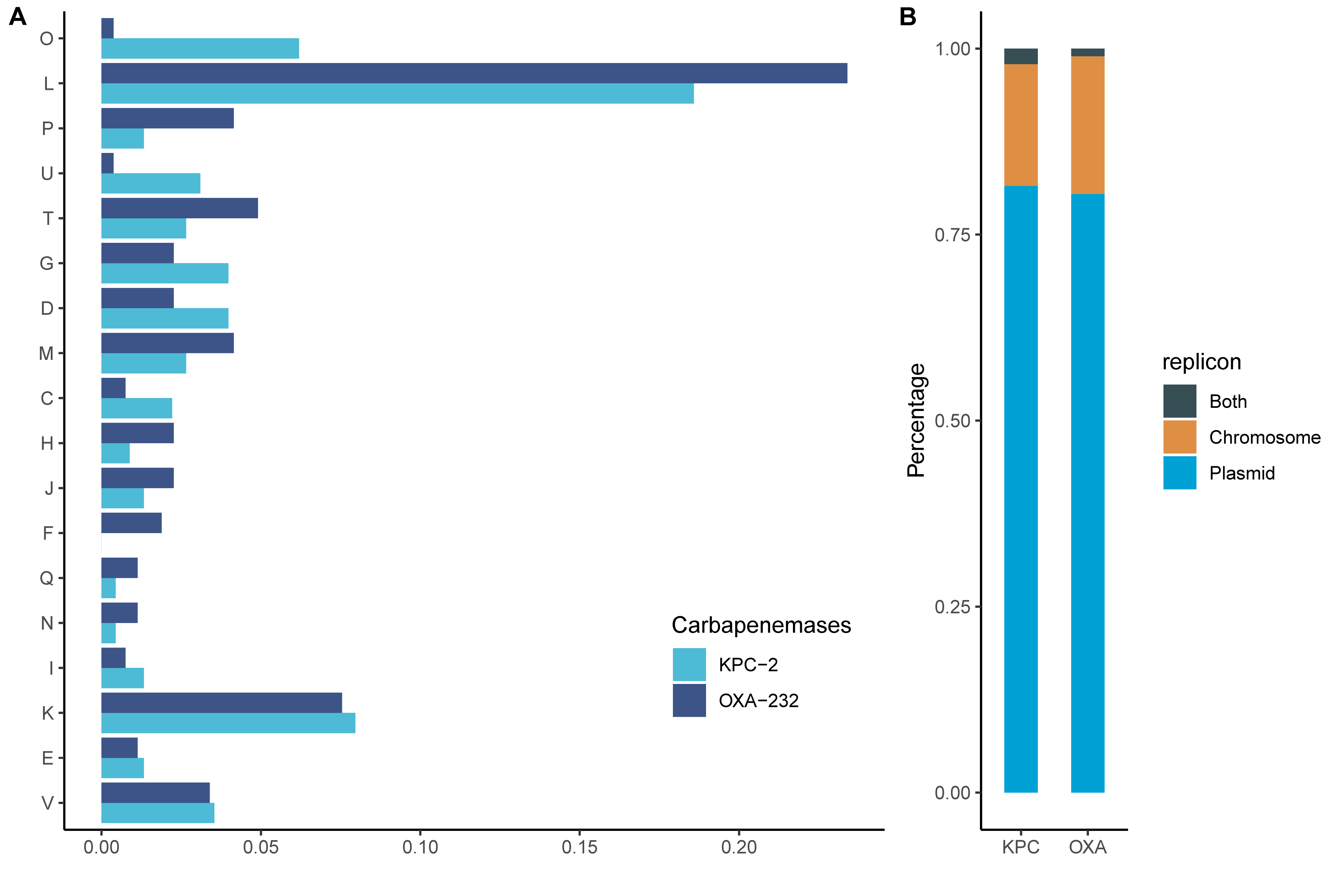

Supplement: Supplementary Figure S5 — COG category annotation (A) and genomic localization (B) statistics of the KPC-2’s and OXA-232’s coincident gene. [file Image_5.TIF]
